# Exercise before breakfast increases 24-h fat oxidation in female subjects

**DOI:** 10.1371/journal.pone.0180472

**Published:** 2017-07-10

**Authors:** Kaito Iwayama, Ryosuke Kawabuchi, Yoshiharu Nabekura, Reiko Kurihara, Insung Park, Masashi Kobayashi, Hitomi Ogata, Momoko Kayaba, Naomi Omi, Makoto Satoh, Kumpei Tokuyama

**Affiliations:** 1 Doctoral Program in Sports Medicine, Graduate School of Comprehensive Human Sciences, University of Tsukuba, Tennodai 1-1-1, Tsukuba, Ibaraki, Japan; 2 International Institute for Integrative Sleep Medicine, University of Tsukuba, Tennodai 1-1-1, Tsukuba, Ibaraki, Japan; University of Birmingham, UNITED KINGDOM

## Abstract

**Background:**

Exercise performed in a postprandial state does not increase 24-h fat oxidation of male and female subjects. Conversely, it has been shown in male subjects that exercise performed in a postabsorptive state increases 24-h fat oxidation compared with that in sedentary control and that with exercise trials performed after breakfast, lunch, or dinner. There is a paucity of study evaluating the effect of exercise performed in a postabsorptive state in female subjects.

**Method:**

Nine young female subjects participated in indirect calorimetry measurement over 24-h using a room-size metabolic chamber in which subjects remained sedentary or performed 60 min exercise before breakfast at 50% of $\dot{V}\;O_{2}\max$. Exercise was accompanied by an increase in energy intake to ensure that subjects were in a similar state of energy balance over 24 h for the two trials.

**Findings:**

Compared with the sedentary condition, exercise performed before breakfast increased 24-h fat oxidation (519 ± 37 vs. 400 ± 41 kcal/day). Time courses of relative energy balance differed between trials with transient negative energy balance observed before breakfast. The lowest values of relative energy balance observed during the 24-h calorimetry, i.e., transient energy deficit, were greater in exercise trials than in sedentary trials. The transient deficit in carbohydrate balance was also observed before breakfast, and magnitude of the deficit was greater in exercise trial compared to that of sedentary trial.

**Interpretation:**

Under energy-balanced conditions, exercise performed in a post-absorptive state increases 24-h fat oxidation in female subjects. The effect of exercise performed before breakfast can be attributed to nutritional state: a transient deficit in energy and carbohydrate at the end of exercise.

## Introduction

Fat oxidation increases during exercise, and its determinants are well-characterized; exercise intensity [1,2], exercise duration [3], training status [4,5], nutritional state [6,7], and gender are the factors affecting fat oxidation during exercise. Fat oxidation during exercise is expected to be increased by prolonged exercise at moderate intensity performed in a post-absorptive state and to be greater in trained individuals and females.

To understand the implications for body weight regulation, the effect of exercise on energy metabolism during the post-exercise recovery period should be considered as well. Several studies evaluated the effect of exercise on fat oxidation over 24 h (24-h fat oxidation) in an energy-balanced condition; as planned by the experimental design, exercise was accompanied by an increase in energy intake to achieve an energy-balanced condition because over- and under-feeding have profound effects on nutrient oxidation [8,9]. Consensus in the literature states that differences in exercise intensity seems to play little role in determining 24-h fat oxidation [10–14].

The effect of exercise on 24-h fat oxidation seems to depend on when it is performed. Exercise performed in a postprandial state does not increase 24-h fat oxidation of male and female subjects [11,12,15,16]. Conversely, we have shown in male subjects that exercise performed in a postabsorptive state, i.e., before breakfast, increase 24-h fat oxidation compared with that in sedentary control and exercise trials performed after breakfast, lunch, or dinner [16–18]. Of note, there is a paucity of study evaluating the effect of exercise performed in a postabsorptive state in female subjects. Physiology and pathophysiology differ between male and female beyond reproductive function [19], and several lines of evidence warrant further studies with female subjects. First, there are gender differences in substrate utilization during and after exercise; females oxidize more fat during exercise, while they oxidize less fat during post-exercise period when compared to males [20–22]. Second, skeletal muscle oxidative activity of males and females responds differently to training. Endurance exercise undertaken in the overnight fasted state was more effective for increasing citrate synthase and 3-hydroxy-CoA dehydrogenase activity in skeletal muscle of males, while exercise after breakfast was more effective in females [23]. Third, there are gender-based differences in content of intermyocellular triacylglycerol (IMTG) and gene expression responsible for fat oxidation. Content of IMTG in female skeletal muscle is higher than that of males [24,25], and female skeletal muscle has abundant protein and mRNA of carnitine palmitoyltransferase 1 [26], FAT/CD36, and hormone sensitive lipase [27] compared to males.

The aim of the present study was to determine whether exercise performed in the postabsorptive state increase 24-h fat oxidation in female subjects. To this end, 24-h indirect calorimetry was performed on two occasions with either a 60 min exercise session before breakfast or sedentary control condition. Both experimental trials were designed to be energy-balanced over 24 h.

## Materials and methods

### Subjects characteristic

Nine young female subjects were recruited to the present study after providing written informed consent. They were moderately physically active, but none of them engaged in endurance training. Subjects had no current medical conditions, and none were taking any medications, including oral contraceptives, at the time of the study. Until one week before the first experiment, subjects spent a night in the metabolic chamber to become familiarized to the measurement condition. This study was approved by the ethics committee of the University of Tsukuba.

### Pre-study evaluation

All subjects performed a graded exercise test comprised of submaximal and maximal tests using a cycle ergometer to determine workload corresponding to 50% of individual maximal oxygen uptake ($\dot{V}O_{2}\max$) [16].

### Experimental protocol

The study was a randomized, repeated measures design comprised of two 24-h calorimetry trials with or without exercise sessions performed before breakfast. To standardize conditions between trials, 24-h calorimetry trials were carried out in the early follicular phase of the menstrual cycle, and all experiments were completed within 2 months. Subjects were asked to maintain their body weight throughout the study, with no significant difference in body weights observed between individual calorimetry trials (P > 0.40).

On the day prior to 24-h calorimetry, subjects entered the metabolic chamber (day 1, 22:00), and 24-h energy expenditure and nutrient oxidation were measured from 6:00 on day 2 to 6:00 on day 3. Once in the metabolic chamber, subjects slept for 7 h from 23:00 to 6:00. On day 2, 3 meals (breakfast at 8:00, lunch at 12:00, and dinner at 18:00) were provided, and subjects exercised at 50% of $\dot{V}O_{2}\max$ for 60 min using a cycle ergometer beginning at 6:30 (exercise trial) or remained in a sedentary position (sedentary trial). Subjects were instructed to remain awake and maintain a sedentary position except when performing prescribed exercise sessions and to sleep only at times specified by the protocol.

Experimental meals were designed to achieve individual energy balance assuming a resting metabolic rate of 22.1 kcal/kg/day according to estimated energy requirements for Japanese individuals [28]. Physical activity factor was assumed to be 1.75 (2,248 ± 65 kcal/day) on day 1, 1.60 (1,991 ± 55 kcal/day) in trials with exercise sessions, and 1.24 in sedentary trials (1,637 ± 42 kcal/day) on day 2. Expressed as percentages of total energy intake, experimental meals consisted of 15% protein, 25% fat, and 60% carbohydrate. The contributions of breakfast, lunch, and dinner to total 24-h energy intake were 33%, 33%, and 34%, respectively.

### Measurements

Energy metabolism was measured using a room-size metabolic chamber (Fuji Medical Science, Chiba, Japan). The airtight chamber measured 2.00 × 3.45 × 2.10 m, with an internal volume of 14.49 m^3^. The temperature and relative humidity of in-coming air was controlled at 25.0°C ± 0.5°C and 55.0% ± 3.0%, respectively. Concentrations of O_2_ and carbon dioxide (CO_2_) in out-going air were measured using an online process mass spectrometer (VG Prima δB, Thermo Electron, Winsford, UK). At every 5 min, O_2_ consumption ($\dot{V}O_{2}$) and CO_2_ production ($\dot{V}{\mathrm{CO}}_{2}$) rates were calculated using an algorithm providing improved transient response [29]. Urine was collected during the indirect calorimetry, and urinary nitrogen concentration was measured using a chemiluminescent nitrogen analyzer (TN-100, Mitsubishi Chemical Corp., Kanagawa, Japan). Macronutrient oxidation and energy expenditure were calculated from $\dot{V}O_{2}$, $\dot{V}{\mathrm{CO}}_{2}$ and urinary nitrogen excretion [30]. Energy and nutrient balance relative to the start of 24-h calorimetry were estimated as the difference between input (meal consumption) and output (oxidation). Relative energy balance was defined as a function of time (t) from 6:00 on day 2.

Relativeenergybalance(t)=accumulatedenergyintake(t)–accumulatedenergyexpenditure(t)

Non-exercise activity was estimated, using a wrist watch-like device (ActiGraph, Ambulatory Monitoring, NY, USA), as the number of times the activity signal crossed the zero reference point per minute [31]. Body composition was measured using the bioimpedance method (BC-118E, TANITA Co., Tokyo, Japan), which estimates fat free mass highly correlated to that determined by dual energy X-ray absorptiometry (r = 0.973)[32].

### Statistical analyses

Data in the main text and figures are presented as means ± SE. Time course of energy expenditure, carbohydrate oxidation and fat oxidation were compared using repeated measures two-way ANOVA for sedentary and exercise trials. Mean values were compared using the paired t-tests for exercise trial and sedentary control trial. Statistical significance was set at P < 0.05. All statistical analyses were performed using SPSS statistical software (Version 22, IBM Japan, Tokyo, Japan).

## Results

Physical characteristics of subjects were 23.9 ± 1.3 year of age, 161.4 ± 1.6 cm of height, 57.8 ± 1.6 kg of body weight and 26.9 ± 1.2% of body fat. Their maximal oxygen uptake ($\dot{V}O_{2}\max$) was 43.6 ± 1.7 ml/kg/min. Habituation of weekly exercise was 2.6 ± 1.0 (h/wk). Work load, relative intensity of the exercise and average heart rate during 60-min exercise session were shown in Table 1. As intended, relative intensity of the exercise was close to 50% of $\dot{V}O_{2}\max$.

**Table 1:** Energy metabolism during exercise.

Work load (W)	87±5
Relative intensity ($\%\dot{V}O_{2}\max$)	52.1±2.9
HR (beats/min)	129±7

**Fig 1:**
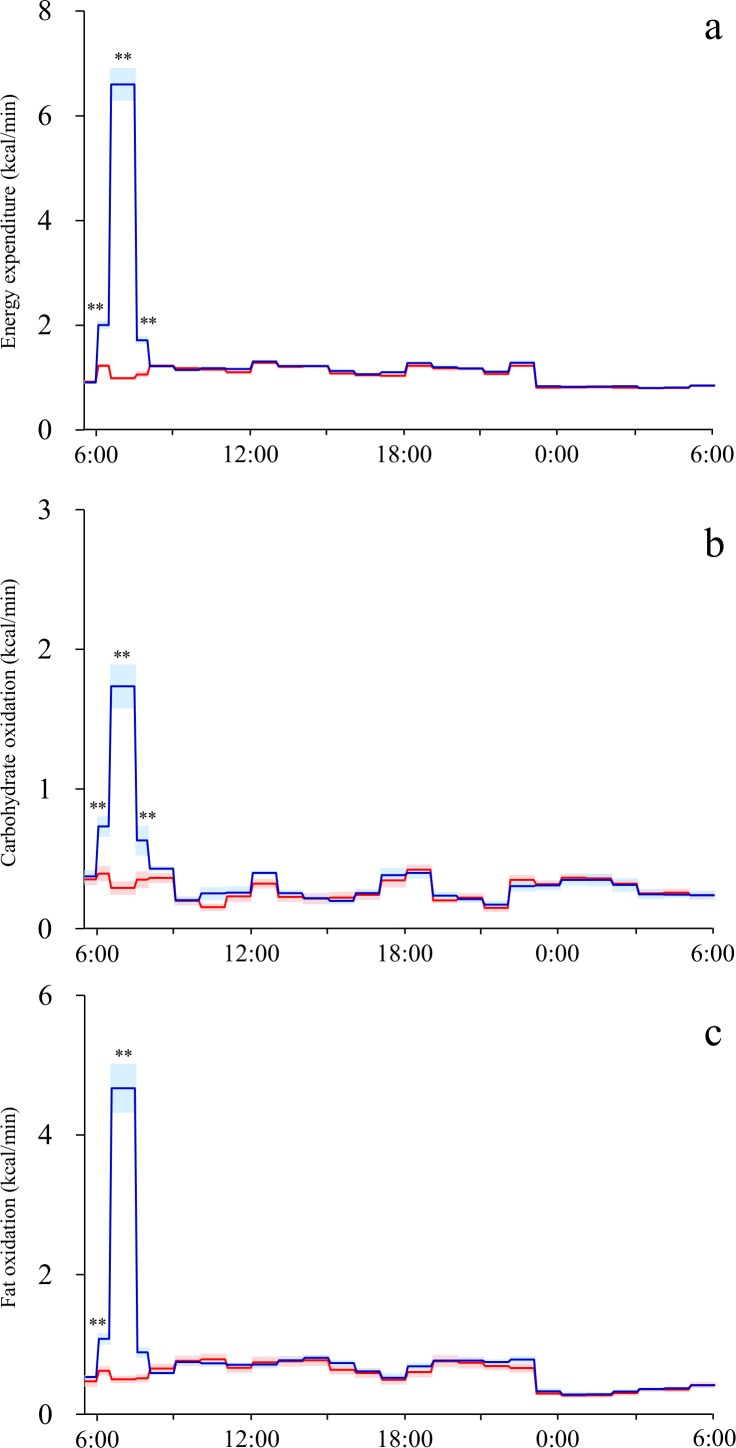
Time course of energy expenditure (a), carbohydrate oxidation (b) and fat oxidation (c) for sedentary (red) and exercise trials (blue). Mean ± SE values plotted at 1-h intervals except for 6:00–6:30 and 7:30–8:00; 6:00–6:30. Significant differences between exercise and sedentary trials: *, P < 0.05; **, P < 0.01.

Time course of energy metabolism during 24 h indirect calorimetry was shown in Fig 1. Compared to the sedentary control trial, energy expenditure and oxidation of carbohydrate and fat were higher during the exercise (6:30–7:30). Prior to exercise (6:00–6:30 due to preparation for exercise) and immediately after exercise (7:30–8:00 due to wiping off sweat and changing clothes), energy expenditure and nutrient oxidation were higher in exercise trials compared to those of control trials, although difference in fat oxidation immediately after the exercise was not statistically significant. Accumulated energy expenditure and carbohydrate and fat oxidation over 24 h were higher for the exercise trial than those of the sedentary trial. No significant differences were observed in 24-h protein oxidation (P = 11) (Table 2).

**Table 2:** Energy metabolism during 24 h.

	Sedentary	Exercise
Energy expenditure (kcal/24h)	1516 ± 40	1925 ± 43*
Carbohydrate oxidation (kcal/24h)	892 ± 35	1126 ± 49*
Fat oxidation (kcal/24h)	400 ± 41	519 ± 37*
Protein oxidation (kcal/24h)	225 ± 34	279 ± 46

*: P < 0.05

**Fig 2:**
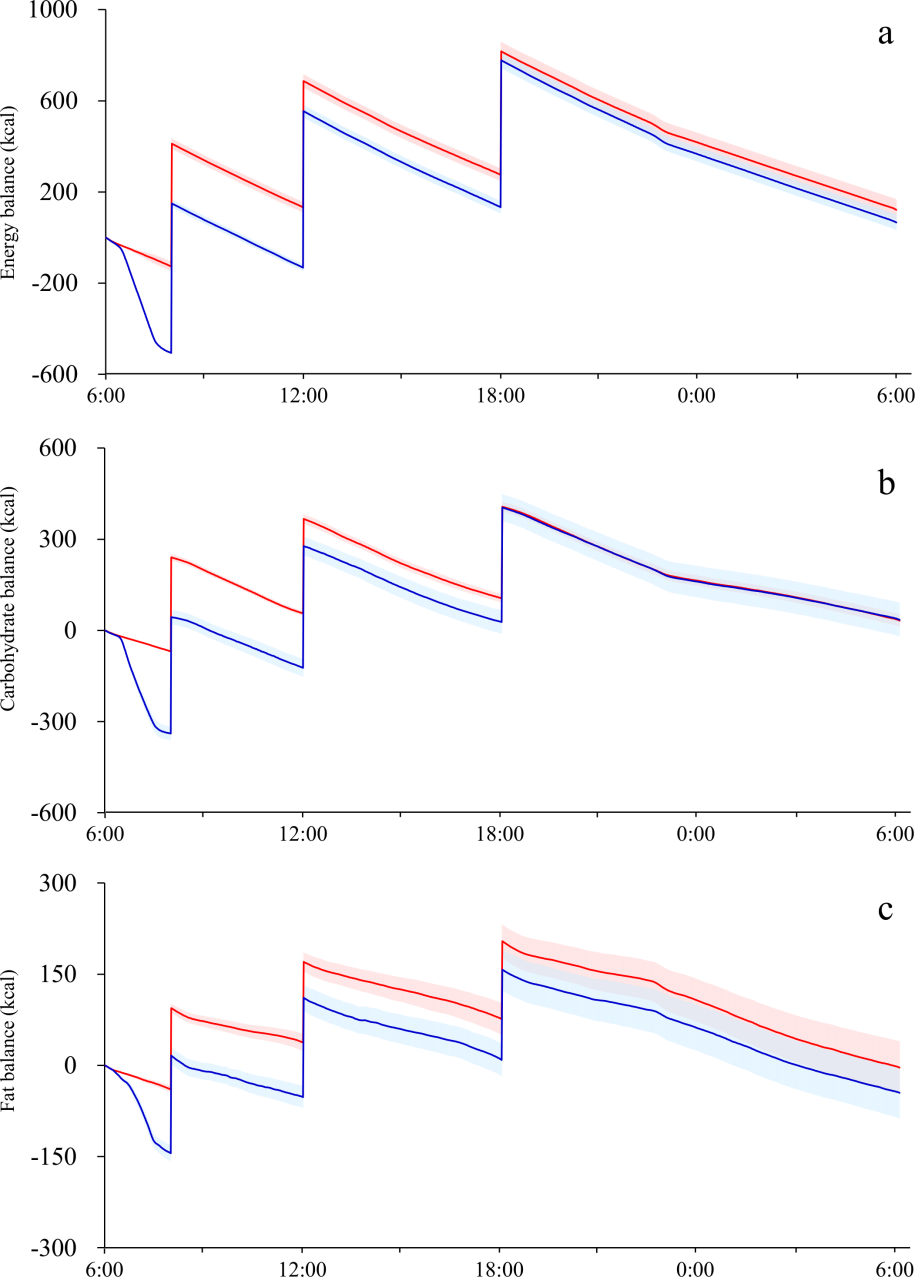
Time course of relative energy balance (a), carbohydrate balance (b) and fat balance (c). Mean ± SE values are plotted at 30-min intervals for exercise trials (blue) and sedentary trials (red).

Time courses of relative energy balance differed between trials with transient negative energy balance observed before breakfast (Fig 2). The lowest values of relative energy balance observed during the 24-h calorimetry, i.e., transient energy deficit, were greater in exercise trials (−507 ± 20 kcal) than in sedentary trials (−127 ± 4 kcal, P<0.01). As planned by experimental design, relative energy balance of both trials eventually converged to a value similar to the initial value by the end of the study (6:00 of the day 3), and there were no significant differences in 24-h energy balance between sedentary (122 ± 34 kcal/day) and exercise trials (67 ± 45 kcal/day, P = 0.20). The transient deficit in carbohydrate balance was also observed before breakfast, and magnitude of the deficit was greater in exercise trial (−339 ± 23 kcal) compared to that of sedentary trial (−69 ± 3 kcal, P<0.01). Similarly, transient deficit in fat balance was also observed (exercise, −144 ± 13; sedentary, −39 ± 6 kcal, P <0.01). Differences in time course of carbohydrate balance between the two experimental conditions gradually decreased, and there was no significant difference at the end of 24 h calorimetry (P = 0.96). Similarly, the differences in time course of relative fat balance became statistically insignificant (P = 0.45).

No significant differences in non-exercise physical activity were observed between sedentary (97 ± 8 counts/min) and exercise trials (105 ± 10 counts/min, P = 0.61).

## Discussion

The main finding of the present study was that exercise performed after overnight fasting increases 24-h fat oxidation in female subjects. Previous studies evaluating in an energy-balanced condition demonstrated that exercise performed in a postprandial state does not increase 24-h fat oxidation in male and female subjects [11,12,15,16]. Conversely, exercise performed before breakfast increased 24-h fat oxidation more than exercise performed after breakfast, lunch, or dinner, or in a sedentary control trial in male subjects [16–18]. Taken together, it is suggested that exercise performed in a postabsorptive state but not that performed in a postprandial state increases 24-h fat oxidation in male and female subjects.

The effect of exercise performed before breakfast may be attributed to the nutritional state or circadian nature of the early morning hours. In our previous studies with male subjects, the effect of exercise performed before breakfast was compared with exercise performed after breakfast, lunch, or dinner, and non-exercise control, and transient energy/carbohydrate deficit was negatively correlated with 24-h fat oxidation [16–18]. In the present study with female subjects, exercise performed in a postabsorptive state induced bigger transient energy/carbohydrate deficit compared with sedentary control trial. Carbohydrate pool size is the smallest among the macronutrients stored in the body, and the metabolic response to changes in carbohydrate storage is more sensitive than the response to fat and protein [8]. Hence, the transient carbohydrate deficit has been indicated as a factor; glycogen shortage induces nuclear translocation of AMPK and upregulation of genes responsible for fat oxidation in skeletal muscle [33], and hepatic glycogen shortage activates a liver–brain–adipose neural axis to enhance lipolysis [34].

It is worth mentioning that additional energy intake for the exercise trials was provided as a proportion of the overall diet as opposed to direct replacement of the substrates oxidized to support exercise. Possibility that mismatch of additional nutrients and those oxidized during the exercise affect 24-h fat oxidation remains to be evaluated. Alternatively, the effect of exercise performed before breakfast can be attributed to the circadian nature of the early morning hours since circadian rhythmicity can be seen in many physiological processes, which include body temperature, activity, sleep, metabolism, and hormone and neurotransmitter secretion [35]. However, the literatures do not support this possibility. First, fat oxidation during exercise in the morning (9:00 h) and evening (17:00 h) was not different when evaluated 3 h after consumption of an identical meal [36]. Second, only a 4-h difference in exercise timing affected 24-h fat oxidation; compared to exercise performed after breakfast (10:30–11:30 h), exercise performed before breakfast (06:30–07:30 h) oxidized significantly more fat [17].

In the present study, the indirect calorimetry was performed in early follicular phase, which is consistent with literature; in many of the metabolic studies with female subjects, follicular phase is often adopted to standardize conditions. It has been reported that fat oxidation during mid-luteal phase is higher compared to early follicular phase, and this is attributed to the difference in the circulating level of sex steroids, such as 17-ß-estradiol [37]. Since glycogen utilization during endurance exercise is influenced by the menstrual cycle [38], an additional study is necessary to assess the effect of exercise on 24-h fat oxidation during luteal phase. In addition, indirect calorimetry itself does not allow us to evaluate sub-spices of substrates: glucose and glycogen for carbohydrate, plasma free fatty acid, triacylglycerol, and IMTG for fat. To understand the metabolic response to exercise performed at different times of day, further studies with other experimental approaches are warranted. For example, insight into tissue metabolites using magnetic resonance spectroscopy or tissue biopsy and/or use of tracers to evaluate substrate kinetics would be valuable.

In terms of the translational potential of the present study, some considerations are required. As a part of the lifestyle diversity in our society, there is wide variation in the time of day that individuals choose to exercise. Recent surveys on time use in the US and Japan have revealed that most people exercise after work, while a few individuals exercise before work on weekdays [39,40]. It requires cautious consideration to decide whether habitual exercise performed in the morning and that performed in the afternoon or early evening have different effects on 24 h fat oxidation. The effect of a single bout of exercise on 24-h fat oxidation cannot be extrapolated to the reduction of body fat with chronic exercise. Compared to training in the fed state, the fasted state suppressed body weight gain from a hypercaloric fat-rich diet in one study in male subjects [41]. However, in other studies with normal diet, no difference in weight change was observed between the training in the fed and fasted state in male and female subjects [23,42–46]. One of the aforementioned studies analyzed weighed food records; average daily intake of energy, protein, and carbohydrate significantly increased during the training, but these increases were not significantly different between the fed and fasted training groups [23]. Evaluating the effect of training performed in fed and fasted state on body composition may require a longer intervention period, which would make it difficult to accurately control or monitor dietary habits during the entire study.

Exercise performed in a postabsorptive state increases 24-h fat oxidation in female subjects. Together with previous studies [11,12,15,16], the present study suggests that effect of single bout of exercise on 24-h fat oxidation depends on when it is performed. Comparison of the chronic effects of exercise performed at different time of day on body fat remains to be performed.

## References

[1] Romijn JA, Coyle EF, Sidossis LS, Gastaldelli A, Horowitz JF, Endert E et al Regulation of endogenous fat and carbohydrate metabolism in relation to exercise intensity and duration. Am J Physiol. 1993; 265: E380–E391. 8214047 10.1152/ajpendo.1993.265.3.E380

[2] Romijn JA, Coyle EF, Sidossis LS, Rosenblatt J, Wolfe RR. Substrate metabolism during different exercise intensities in endurance-trained women. J Appl Physiol. 2000; 88: 1807–1714. 10.1152/jappl.2000.88.5.1707 10797133

[3] Coyle EF. Substrate utilization during exercise in active people. Am J Clin Nutr. 1995; 61: 968S–79S. 7900696 10.1093/ajcn/61.4.968S

[4] Nordby P, Saltin B, Helge JW. Whole-body fat oxidation determined by graded exercise and indirect calorimetry: a role for muscle oxidative capacity? Scand J Med Sci Sports. 2006; 16: 209–214. doi: 10.1111/j.1600-0838.2005.00480.x 16643200 10.1111/j.1600-0838.2005.00480.x

[5] Stisen AB, Stougaard O, Langfort J, Helge JW, Sahlin K, Madsen K. Maximal fat oxidation rates in endurance trained and untrained women. Eur J Appl Physiol. 2006; 98: 497–506. doi: 10.1007/s00421-006-0290-x 17006714 10.1007/s00421-006-0290-x

[6] Horowitz JF, Mora-Rodriguez R, Byerley LO, Coyle EF. Substrate metabolism when subjects are fed carbohydrate during exercise. Am J Physiol. 1999; 276: E828–E835. 10329975 10.1152/ajpendo.1999.276.5.E828

[7] Wallis GA, Dawson R, Juul Achten, Webber J, Jeukendrup AE. Metabolic response to carbohydrate ingestion during exercise in males and females. Am J Physiol. 2006; 290: E708–E715. 10.1152/ajpendo.00357.2005 16278245

[8] Flatt JP. Importance of nutrient balance in body weight regulation. Diabetes Metab Rev. 1988; 4: 571–581. 3065010 10.1002/dmr.5610040603

[9] Horton TJ, Drougas H, Brachey A, Reed GW, Peters JC, Hill JO. Fat and carbohydrate overfeeding in humans: different effects on energy storage. Am J Clin Nutr. 1995; 62: 19–29. 7598063 10.1093/ajcn/62.1.19

[10] Melanson EL, Sharp TA, Seagle HM, Horton TJ, Donahoo WT, Grunwald GK et al Effect of exercise intensity on 24-h energy expenditure and nutrient oxidation. J Appl Physiol. 2002; 92: 1045–1052. doi: 10.1152/japplphysiol.00706.2001 11842038 10.1152/japplphysiol.00706.2001

[11] Melanson EL, Gozansky WS, Barry DW, MacLean PS, Grunwald GK, Hill JO. When energy balance is maintained, exercise does not induce negative fat balance in lean sedentary, obese sedentary, or lean endurance-trained individuals. J Appl Physiol. 2009a; 107: 1847–1856. 19833807 10.1152/japplphysiol.00958.2009 PMC3774345

[12] Melanson EL, MacLean PS, Hill JO. Exercise improves fat metabolism in muscle but does not increase 24-h fat oxidation. Exerc Sport Sci Rev. 2009b; 37: 93–101. 19305201 10.1097/JES.0b013e31819c2f0b PMC2885974

[13] Saris WHM, Schrauwen P. Substrate oxidation differences between high- and low-intensity exercise are compensated over 24 hours in obese men. Int J Obesity. 2004; 28: 759–765. 10.1038/sj.ijo.0802631 15052277

[14] Treuth MS, Hunter GR, Williams M. Effects of exercise intensity on 24-h energy expenditure and substrate oxidation. Med Sci Sports Exerc. 1996; 28: 1138–1143. 8883001 10.1097/00005768-199609000-00009

[15] Dionne I, Van Vugt S, Tremblay A. Postexercise macronutrient oxidation: a factor dependent on postexercise macronutrient intake. Am J Clin Nutr. 1999; 69: 927–930. 10232632 10.1093/ajcn/69.5.927

[16] Iwayama K, Kurihara R, Nabekura Y, Kawabuchi R, Park I, Kobayashi M et al Exercise increases 24-h fat oxidation only when it is performed before breakfast. EBioMed. 2015a; 2: 2003–2009. 10.1016/j.ebiom.2015.10.029 PMC4703705 26844280

[17] Shimada K, Yamamoto Y, Iwayama K, Nakamura K, Yamaguchi S, Hibi M et al Effect of exercise performed before or after breakfast on 24-h fat oxidation. Metabolism. 2013; 62: 793–800. doi: 10.1016/j.metabol.2012.12.008 23313101 10.1016/j.metabol.2012.12.008

[18] Iwayama K, Kawabuchi R, Park I, Kurihara R, Kobayashi M, Hibi M et al Transient energy deficit induced by exercise increases 24-h fat oxidation in young trained men. J Appl Physiol. 2015b; 118: 80–85. 25554797 10.1152/japplphysiol.00697.2014

[19] Miller VM. In pursuit of scientific excellence: sex matters. J Appl Physiol. 2012; 112: 1427–1428. doi: 10.1152/japplphysiol.00303.2012 22422798 10.1152/japplphysiol.00303.2012

[20] Horton TJ, Pagliassotti MJ, Hobbs K, Hill JO. Fuel metabolism in men and women during and after long-duration exercise. J Appl Physiol. 1998; 85: 1823–1832. 9804587 10.1152/jappl.1998.85.5.1823

[21] Henderson GC, Fattor JA, Horning MA, Faghihnia N, Johnson ML, Mau TL et al Lipolysis and fatty acid metabolism in men and women during the postexercise recovery period. J Physiol. 2007; 584: 963–981. doi: 10.1113/jphysiol.2007.137331 17855762 10.1113/jphysiol.2007.137331 PMC2277001

[22] Henderson GC, Alderman BL. Determinants of resting lipid oxidation in response to a prior bout of endurance exercise. J Appl Physiol. 2014; 116: 95–103. doi: 10.1152/japplphysiol.00956.2013 24235102 10.1152/japplphysiol.00956.2013

[23] Stannard SR, Buckley AJ, Edge JA, Thompson MW. Adaptations to skeletal muscle with endurance exercise training in the acutely fed versus overnight-fasted state. J Sci Med Sport. 2010; 13: 465–469. doi: 10.1016/j.jsams.2010.03.002 20452283 10.1016/j.jsams.2010.03.002

[24] Roepstorff C, Donsmark M, Thiele M, Vistisen B, Stewart G, Vissing K et al Sex differences in hormone-sensitive lipase expression, activity and phosphorylation in skeletal muscle at rest and during exercise. Am J Physiol. 2006; 291: E1106–E1114. 10.1152/ajpendo.00097.2006 16822962

[25] Tarnopolsky MA, Rennie CD, Robertshaw HA, Fedak-Tarnopolsky SN, Devries MC, Hamadeh MJ. Influence of endurance exercise training and sex on intramyocellular lipid and mitochondrial ultrastructure, substrate use and mitochondrial enzyme activity. Am J Physiol. 2007; 292: R1271–R1278. 10.1152/ajpregu.00472.2006 17095651

[26] Berthon PM, Howlett RA, Heigenhauser GJF, Spriet LL. Human skeletal muscle carnitine palmitoyltransferase I activity determined in isolated intact mitochondria. J Appl Physiol. 1998; 85: 148–153. 9655768 10.1152/jappl.1998.85.1.148

[27] Kiens B, Roepstorff C, Glatz JFC, Bonen A, Schjerling P, Knudsen J et al Lipid-binding proteins and lipoprotein lipase activity in human skeletal muscle: influence of physical activity and gender. J Appl Physiol. 2004; 97: 1209–1218. doi: 10.1152/japplphysiol.01278.2003 15155715 10.1152/japplphysiol.01278.2003

[28] Anon. Dietary reference intakes for Japanese. Ministry of Health Labour and Welfare of Japan, Tokyo 2010.

[29] Tokuyama K, Ogata H, Katayose Y, Satoh M. Algorithm for transient response of whole body indirect calorimeter: deconvolution with a regularization parameter. J Appl Physiol. 2009; 106: 640–650. doi: 10.1152/japplphysiol.90718.2008 19008487 10.1152/japplphysiol.90718.2008

[30] Ferrannini E. The theoretical basis of indirect calorimetry: A review. Metabolism. 1988; 37: 287–301. 3278194 10.1016/0026-0495(88)90110-2

[31] Sato M, Nakamura K, Ogata H, Miyashita A, Nagasaka S, Omi N et al Acute effect of late evening meal on diurnal variation of blood glucose and energy metabolism. Obesity Research & Clinical Practice. 2011; 5: e220–e228 10.1016/j.orcp.2011.02.001 24331104

[32] Naka T, Han I, Keii T et al Body composition evaluated by segmental bioelectrical impedance analysis in healthy subjects and athletes. Jpn J Phys Fitness Sports Med. 2006; 55 (Suppl), S49–S52.

[33] Philp A, Hargreaves M, Baar K. More than a store: regulatory roles for glycogen in skeletal muscle adaptation to exercise. Am J Physiol. 2012; 302: E1343–E1351. 10.1152/ajpendo.00004.2012 22395109

[34] Izumida Y, Yahagi N, Takeuchi Y, Nishi M, Shikama A, Takarada A et al Glycogen shortage during fasting triggers liver–brain–adipose neurocircuitry to facilitate fat utilization. Nature Comun. 2013; 4: 2316. 10.1038/ncomms3316 PMC3753545 23939267

[35] Hastings MH, Maywood ES, Reddy AB. Two decades of circadian time. J Neuroendocrinol. 2008; 20: 812–819. doi: 10.1111/j.1365-2826.2008.01715.x 18601704 10.1111/j.1365-2826.2008.01715.x

[36] Kim HK, Ando K, Tabata H, Konishi M, Takahashi M, Nishimaki M et al Effects of different intensities of endurance exercise in morning and evening on the lipid metabolism response. J Sports Sci Med. 2016; 15: 467–476. 27803625 PMC4974859

[37] Tarnopolsky MA. Sex differences in exercise metabolism and the role of 17-beta estradiol. Med Sci Sports Exerc. 2008; 40: 648–654. doi: 10.1249/MSS.0b013e31816212ff 18317381 10.1249/MSS.0b013e31816212ff

[38] Devries MC, Hamadeh MJ, Phillips SM, Tarnopolsky MA. Menstrual cycle phase and sex influence muscle glycogen utilization and glucose turnover during moderate-intensity endurance exercise. Am J Physiol. 2006; 291: R1120–R1128. 10.1152/ajpregu.00700.2005 16690766

[39] Anon. Sports and Exercise. Bureau of Labor Statistics. United States Department of Labor. Available from http://www.bls.gov/spotlight/2008/sports/, Accessed 2 August 2015.

[40] Anon. Survey on Time Use and Leisure Activities. Statistics Bureau, Ministry of Internal Affairs and Communications, Japan, Tokyo 2013. Accessed 2 August 2015.

[41] Van Proeyen K, Szlufcik K, Nielens H, Pelgrim K, Deldicque L, Hesselink M et al Training in the fasted state improves glucose tolerance during fat-rich diet. J Physiol. 2010; 588: 4289–4302. doi: 10.1113/jphysiol.2010.196493 20837645 10.1113/jphysiol.2010.196493 PMC3002457

[42] De Bock K, Derave W, Eijnde BO, Hesselink MK, Koninckx E, Rose AF et al Effect of training in the fasted state on metabolic response during exercise with carbohydrate intake. J Appl Physiol. 2008; 104: 1045–1055. doi: 10.1152/japplphysiol.01195.2007 18276898 10.1152/japplphysiol.01195.2007

[43] Gillen JB, Percival ME, Ludzki A, Tarnopolsky MA, Gibala MJ. Interval training in the fed or fasted state improves body composition and Muscle oxidative capacity in overweight women. Obesity. 2013; 21: 2249–2255. doi: 10.1002/oby.20379 23723099 10.1002/oby.20379

[44] Nybo L, Pedersen K, Christensen B, Aagaard P, Brandt N, Kiens B. Impact of carbohydrate supplementation during endurancetraining on glycogen storage and performance. Acta Physiol. 2009; 197: 117–127. 10.1111/j.1748-1716.2009.01996.x 19432594

[45] Schoenfeld BJ, Aragon AA, Wilborn CD, Kringer JW, Sonmez GT. Body composition changes associated with fasted versus non-fasted aerobic exercise. J Int Soc Sports Nutr. 2014; 11: 54 doi: 10.1186/s12970-014-0054-7 25429252 10.1186/s12970-014-0054-7 PMC4242477

[46] Van Proeyen K, Szlufcik K, Nielens H, Ramaekers M, Hespel P. Beneficial metabolic adaptations due to endurance exercise training in the fasted state. J Appl Physiol. 2011; 110: 236–245. doi: 10.1152/japplphysiol.00907.2010 21051570 10.1152/japplphysiol.00907.2010 PMC3253005

